# Pregnancy following successful accelerated rescue oocyte retrieval following absence of oocytes in primary follicular aspirations: A case report

**DOI:** 10.1097/MD.0000000000043117

**Published:** 2025-07-18

**Authors:** Feras Sendy, Robert Hemmings

**Affiliations:** a Ovo Fertility Clinic, Reproductive Endocrinology and Infertility, Montreal, Quebec, Canada; b Department of Obstetrics and Gynecology, University of Montreal, Montreal, Quebec, Canada; c Department of Obstetrics and Gynecology, King Fahad Medical City, Riyadh, Saudi Arabia.

**Keywords:** Embryo transfer, empty follicle syndrome, IVF, pregnancy, rescue trigger

## Abstract

**Rationale:**

The objective of this study was to present a case of successful oocyte retrieval following a failed oocyte pickup on the same day that led to pregnancy after frozen embryo transfer.

**Patient concerns:**

A 31-year-old gravida 1 para 1 woman with Von Willebrand disease, presented to our infertility center for secondary infertility for 2 years with known male factor and failed initial oocyte collection.

**Diagnoses:**

Increasing progesterone, decreasing estradiol, and low luteinizing hormone were signs of a successful ovulation and indicated the need to perform rescue retrieval.

**Interventions:**

In vitro fertilization, rescue delayed oocyte retrieval, and letrozole stimulated frozen embryo transfer.

**Outcomes:**

A pregnancy following a frozen embryo transfer after a rescue oocyte retrieval in a stimulated in vitro fertilization cycle. Interpretation of blood tests (estradiol, progesterone, and luteinizing hormone) suggestive of effective ovulation induction with an agonist trigger can be an indication for delayed rescue retrieval.

**Lessons:**

Despite the absence of oocyte after 5 follicular aspiration, healthcare providers should be aware of the value of rescue oocyte collection when endocrine findings suggest an effective ovulation trigger.

## 1. Introduction

Empty follicle syndrome (EFS) is defined as the absence of oocytes after aspiration of mature follicles.^[1]^ It occurs in up to 3.5% of in vitro fertilization cycles.^[2,3]^ EFS risk factors includes low baseline luteinizing hormone, high or low body mass index, prolonged use of contraception and long duration of ovarian stimulation.^[4,5]^

Here, we report a case of successful rescue oocyte retrieval after failure to obtain oocytes in 5 primary follicular aspiration that led to pregnancy following a frozen embryo transfer.

## 2. Case report

A 31-year-old gravida 1 para 1 woman presented to our infertility center for secondary infertility of 2 years duration associated with a male factor. She had a body mass index of 19.3. Her past medical history included Von Willebrand disease (VWD). She had regular mensural cycles, a normal ovarian reserve with an anti-mullerian hormone of 2.40 ng/mL and antral follicle count of 23. Her partner was a 40-year-old male diagnosed with teratospermia. The tunnel test for deoxyribonucleic acid fragmentation was at 20%. The patient had 4 failed cycles of intrauterine insemination (3 with letrozole 5 mg alone and 1 with letrozole 5 mg and Gonal-F^®^ 37.5 IU/mL). She subsequently underwent ovarian stimulation with an antagonist protocol (Menopur^®^ 300 UI/mL/, Rekovelle^®^ 12 mcg/Cetrotide^®^ 250 mcg) and agonist trigger alone with Suprefact 1 mg/mL. The day of trigger, she had 26 follicles at ≥ 14mm. Her estradiol and progesterone were at 20,783 pmol/L and 7.02 nmol/L, respectively. The patient took 1500 mg of tranexamic acid^®^ and desmopressin^®^ 18 mcg 2 hours and 1 hour prior to oocyte pickup respectively to reduce the risk of bleeding. Furthermore, a transvaginal ultrasound was performed prior to oocyte pickup to ensure the presence of follicles. The oocyte pickup was done 37 hours after the agonist trigger. No oocytes were found after 5 follicular aspirations. Blood was then drawn for estradiol, progesterone, and luteinizing hormone and showed the following results: 9914 pmol/L, 42.45 nmol/L, and 1.47 IU/L, respectively. A second oocyte pickup was performed 4 hours and half later. Twenty oocytes were obtained. Fourteen were mature for in vitro fertilization after sperm preparation with microfluidic sperm selection (ZyMot™). Thirteen were fertilized, 11 reached day 5 blastocyst stage, and were vitrified. Frozen transfer of 1 day 5 embryo grade 2AA was done on a stimulated cycle with letrozole. Luteal phase support was done with vaginal progesterone of 300 mg daily. Her 1st human chorionic gonadotropin 11 days post transfer was 242 mIU/mL, followed by 557 mIU/mL 48 hours later. Therefore, she was advised to continue progesterone up to 12 weeks of gestation. The transvaginal ultrasound found an intrauterine gestational sac with a positive yolk sac, fetal pole, and a positive fetal cardiac activity, with a crown rump length corresponding to 7 weeks and 2 days (7 weeks and 1 day according to the day of embryo transfer). All her human chorionic gonadotropin blood tests were done in the same laboratory. A follow up transvaginal ultrasound at 8 weeks of gestation in our center showed adequate fetal growth. The pregnancy evolution subsequently was normal.

## 3. Discussion

This is the first reported case of a successful rescue oocyte retrieval after failure to obtain oocytes in 5 primary follicular aspiration leading to a viable pregnancy after frozen blastocyst transfer. No prior case in the literature describes such a procedure. This underlines the unique nature of this case and its potential to challenge existing framework on empty follicle syndrome.

Due to the risk of bleeding with VWD, we gave the patient desmopressin^®^ and tranexamic acid^®^ prior to oocyte retrieval. VWD patients are known to have an increased risk of bleeding.^[6]^ However, we did not find a correlation between failed oocyte collection and VWD in the literature. It is possible that bleeding may complicate oocyte pickup as the presence of clots could block the passage of oocytes from needle. Theoretically, using the prescribed drugs could increase the clotting leading to blood clot obstructing the aspiration needle. Our primary failed oocyte collection did not have abnormal bleeding or clotting leading to difficulty in the retrieval process. The absence of oocytes was confirmed by 2 embryologists.

Our patient had a body mass index of 19.3 which is a known risk factor of empty follicle syndrome.^[4]^ On the other hand, her blood tests after the 1st oocyte pickup were in favor of effective ovulation (a decreasing estradiol of 9914 pmol/L compared to 20,783 pmol/L, and a high progesterone of 42.45 nmol/L compared to 7.02 nmol/L) that led us to perform a rescue oocyte collection on the same day. A study indicated that a progesterone <11.13 nmol/L 8 to 12 hours post trigger was associated with a failed trigger using gonadotropin releasing hormone agonist.^[7]^ Although we did not perform the blood tests 8 to 12 hours post agonist trigger yet we had a progesterone of 42.45 nmol/L the day of oocyte pickup confirming ovulation.

The time interval between ovulation induction and oocyte retrieval is crucial, as it can significantly affect outcomes. Variations in this timing, especially if oocyte retrieval occurs outside the optimal range, may lead to changes in results or even empty follicle syndrome.^[8]^ For instance, it has been reported that the optimal time interval for antagonist protocol is 34.5 to 36.3 hours post agonist trigger for adequate mature oocyte retrieval. Despite the proper timing and dose of medication administration with our patient no oocytes were found after 5 mature follicular aspirations. The time interval in our case for the 1st and rescue oocyte collection was 37 hours and 41 hours and 30 minutes, respectively. The delay for performing the second retrieval 4 and a half hours later was caused by the delay in obtaining laboratory results and the return of the patient to the clinic.

In our clinic, the fertility specialist usually stops the oocyte retrieval if the embryologist confirms the absence of oocyte after 3 to 5 follicular aspirations. Certain practices terminate oocyte retrieval after failure to obtain oocytes from 1 ovary.^[9]^

This case report fills 2 main gaps in EFS clinical practice:

First, in the current literature so far there is no evidence that an emergency oocyte retrieval will succeed a few hours after a first failed aspiration when hormone measurements indicate that ovulation has occurred.

Second, there is no data on the effect of VWD and the use of hemostatic agents on the process of oocyte aspiration.

This case does not represent a true EFS since the true condition of EFS occurs when oocytes disappear completely while patients meet follicular development demands alongside proper trigger administration and when ovarian dysfunction or drug failure cause this effect.^[10]^ False EFS develops because of drug malabsorption or problems with retrieval techniques. The post-first-retrieval hormonal analysis indicated ovulation process had started because estradiol plummeted while progesterone levels rose. Genuine EFS would not allow oocyte retrieval in a rescue retrieval yielding 20 oocytes. The clinical manifestations initially matched EFS but biochemistry tests showed that this case was not an EFS.

This case report suggests that rather than concluding an empty follicle syndrome, rescue retrieval 4 hours after a failed first retrieval if endocrine findings confirm proper ovulation trigger might be successful and might offer patients a chance at achieving a clinical pregnancy.

This case suggests that for some patients the time interval between ovulation induction and oocyte retrieval can be as long as 41 and a half hours when endocrine results confirm proper ovulation trigger.

In conclusion, this report is relevant to assisted reproductive technologies providers. It can be generalized to patients with adequate evidence of ovulation and failed oocyte retrieval under the condition that a remaining number of follicles have not been aspirated.

## Authors contribution

**Supervision:** Robert Hemmings.

**Validation:** Feras Sendy.

**Visualization:** Feras Sendy.

**Writing – original draft:** Feras Sendy.

**Writing – review & editing:** Feras Sendy, Robert Hemmings.
